# “Stories like ours continue”: A phenomenological exploration of the lived experiences of ex-serial clinical trial volunteers in South India

**DOI:** 10.1016/j.dialog.2025.100256

**Published:** 2025-11-07

**Authors:** Irene Sambath, Kalpana B. Kosalram

**Affiliations:** SRM School of Public Health, SRM Institute of Science & Technology, Kattankulathur, Chengalpattu, Tamil Nadu 603203, India

**Keywords:** Serial volunteering, Clinical trials, Interpretative phenomenological analysis, Lived experiences, Qualitative study

## Abstract

**Purpose:**

Indian media has frequently highlighted the plight of vulnerable low-income groups who over-volunteer in clinical trials to make ends meet, often at the cost of their health. Despite these revelations, the lived experiences of these individuals remain insufficiently documented in academic literature. This study seeks to address this gap by exploring how clinical trial volunteers construct their world around their experiences of having taken part in multiple trials over a span of time. Additionally, the study aims to shed light on the current circumstances and challenges faced by these individuals and their families.

**Methods:**

Using Interpretative Phenomenological Analysis, this study analyses in-depth interviews conducted using respondent-driven sampling. Participants include three ex-serial volunteers who discontinued clinical trial participation and three key informants of deceased serial trial volunteers, from a remote mandal in the state of Telengana, South India.

**Results:**

Three superordinate themes emerged: *A journey of strain and stain, Transcending all turmoil to remain acceptable to system and society*, and *Agony of the family*, each with five subthemes. The personal narratives highlight how financial desperation coerced them into serial participation. Over time, repeated enrolment led to physical and emotional deterioration, concealment, stigma, and family distress. Families discovered trial involvement only after adverse events or death, highlighting systemic gaps in transparency and post-trial care.

**Conclusion:**

Despite existing government regulations, the underground nature of recruitment networks and the persistent demand for willing participants enable these practices to thrive. Findings underscore the urgent need for a national registry, stronger oversight, and community-level awareness to ensure ethical, scientifically sound, and socially responsible clinical research in India.

## 1 Introduction

Clinical trials are the cornerstone of global drug development, serving as a vital process to test the safety and efficacy of new medicines and vaccines before they are approved for widespread use [1]. The drug discovery process spans multiple phases, involving preclinical testing on animals, followed by clinical trials with patients and healthy volunteers [1]. Worldwide, they are governed by ethical frameworks such as the Declaration of Helsinki [2] and the Council for International Organizations of Medical Sciences (CIOMS) Guidelines [3]. Despite these safeguards, disparities in life circumstances and economic status continue to expose vulnerable populations to exploitation in the name of scientific progress. The globalization of clinical research over the past decades has led to a marked shift in the location of early-phase and bioequivalence studies to low- and middle-income countries (LMICs). As articulated by Rajan's (2007) [4] “biocapital” and “surplus health” in India's experimental economies, India has become a prominent hub for conducting clinical research for a number of factors: easy availability of diversified genetic pool of population, skilled professionals, advanced technological capabilities, cost effective location for conducting trials, low human resource, low recruitment cost, lower compensation rates, expedited regulatory approval, presence of dual burden of communicable and non-communicable, which provide a range of conditions for research study [[4], [5], [6]].

In India, Clinical Research Organizations (CROs), working on behalf of pharmaceutical companies, play a significant role in recruiting participants for clinical trials, particularly bioequivalence studies that involve healthy volunteers [7,8]. These bioequivalence studies, which test the metabolism of generic drugs, are seen as relatively safer compared to early-phase trials [9]. Notably, in resource-limited settings like India, most healthy volunteers come from socio-economically disadvantaged backgrounds, with low literacy levels, and limited access to information [10]. Discussions around paid research subjects have traditionally categorized them into: “students, who participate for extra spending money; low-income unskilled workers, who participate for the small financial remuneration; and professional ‘guinea pigs’ who chose to participate as a way of maintaining a marginal lifestyle” as reckoned by Zink (2001) (p. 88) [11].

Qualitative studies from the USA [[12], [13], [14]], Australia [15], essay by Tishler & Bartholomae (2003) [14] and existing studies from India [[16], [17], [18], [19], [20]], including a meta-analysis of qualitative studies [21] suggest that financial desperation is the prime factor for participation, while awareness and knowledge about clinical trials remain low or incomplete among the general public and patient populations. Similarly, Abadie (2010) [22] and Mwale (2017) [23] in their books have documented sociological and ethnographic accounts of how healthy volunteers use clinical trials to supplement their income. Whereas motivations are varied for healthy participants from a study in Australia [24] and that they were driven by factors other than money.

Indian media has frequently highlighted the plight of vulnerable low-income groups who over-volunteer in clinical trials to make ends meet, often at the cost of their health and well-being [25]. Despite these repeat volunteerism revelations, the lived experiences of these individuals remain insufficiently documented in the academic literature. The study does not seek to evaluate the ethical and regulatory framework of the clinical trial system in India; rather, it seeks to illuminate the human realities that unfold within it. By capturing the personal narratives of serial clinical trial volunteers and their families, this study seeks to understand how these individuals construct their world around their experiences of having taken part in multiple trials over a span of time, how they negotiate risk and survival, and navigate the moral and social implications of their choices. The focus is on amplifying their voices and experiences and contribute to broader discussions on healthy volunteering in clinical trials.

## 2 Methodology

### 2.1 Qualitative approach

We utilized the Interpretative Phenomenological Analysis (IPA) [26,27], a qualitative research approach rooted in phenomenology, hermeneutics and idiography, to understand the lived experiences of serial clinical trial volunteers. The primary aim of IPA is to explore how participants make sense of their lived experiences and their perceptions of the world. Unlike other research methods that emphasize objectivity, IPA is concerned with subjective, conscious appraisals of the experiences. IPA acknowledges that an individual's perceptions are shaped by their personal ideas, judgements, and interpretations. In this approach, the ‘active’ role of the researcher is acknowledged in accessing the ‘life-world’ of the participant. This is a double hermeneutic process, where the participant is making sense of their experience, and the researcher, in turn, interprets the participants interpretations. This layered process inherently limits access to a purely non-interpretative account of the participant's experiences. The researcher's own perspectives, experiences, and presuppositions are carefully correlated throughout the process, as they simultaneously seek deeper insights by reflecting on and evaluating their interpretations.

### 2.2 Study setting and participant approach

The selection of the study setting was guided by initial news reports of a deceased clinical trial volunteer from a remote mandal in the state of Telengana, South India. ‘*Mandal*’ is a term used in India to denote a local administrative area made of towns and villages. IS followed the case, visited the family to verify the facts, explained and assessed the feasibility of conducting further research in the region. During this preliminary visit, the family provided consent for a follow-up interview, allowing for a more in-depth exploration of their experiences. This interaction, along with the prevalence of clinical trial participation in the area, led to the finalization of the remote mandal in Telangana as the study setting.

### 2.3 Sampling and participant recruitment

As noted by Smith et al. (2009), IPA studies have been published with small number of participants (typically more than three), enabling the researcher to capture a detailed and nuanced interpretative account of the responses, prioritizing depth for breadth [26]. The first participant recruited for this study was a family member of a deceased volunteer whose story made headlines in local news channels and leading national newspapers in India. Subsequent participants were identified through respondent-driven sampling, initiated by the first participant, and snowballed to include the remaining individuals in the samples. Participants in this study are three ex-clinical trial volunteers who had discontinued participation in trials, and three key informants of deceased serial trial volunteers, all from remote villages from a mandal in Telengana. None of the participants approached declined to participate in the study. This study did not follow data saturation due to the hard-to-reach nature of the participant population. The recruitment and data collection process spanned from June 2019 to January 2020, after which the onset of the COVID-19 pandemic further restricted fieldwork. Given these constraints, the study was limited to six participants.

### 2.4 Ethical considerations and data collection

Due to the sensitive nature of the topic, participants chose to be interviewed at their homes, in the presence of family members, and in Telugu, their native language, to ensure comfort and open expression. Each interview was conducted at least two days apart. IS conducted the interviews with the assistance of a village representative fluent in both Telugu and English, who facilitated communication when needed. The In-depth Interview Guide in English and Telugu (Supplementary Material S1 and S2) served as a reference rather than a rigid framework, allowing for a natural and unrestricted flow of conversation. Verbal consent was obtained, and detailed contemporaneous narrative scripts were taken during the interviews, as audio recordings were not used to respect the participants' preferences. This approach allowed for the capture of key expressions and emotions. Such field-note-based documentation has been employed in sensitive qualitative research where recording may inhibit disclosure [28]. Participants were assured that their responses would remain confidential and their identities anonymous. Interviews lasted between forty-five minutes to one hour, with additional time for participants to share further reflections until they felt they had nothing more to say. No financial or material incentives were provided to participants for their participation in this study. The study was approved by the Institutional Review Board of SRM School of Public Health, SRM Institute of Science & Technology, Tamil Nadu, India. Reporting followed the COREQ checklist (Supplementary Table S3) [29].

### 2.5 Data analysis

The scripts were analysed manually using IPA by IS. The accounts of the participants were read and re-read to become familiar with the data. The right-hand margin was used to annotate explanatory notes on interesting or significant points related to the participants' experiences. Having completed this, the left-hand margin was used to document emerging theme titles. These theme titles were generated from the initial notes on the right-hand margin to concise phrases retaining the quality of what was found in the transcript. They were then grouped or clustered by connections as they were perceived by the researcher. At this stage, the clustered themes were checked against the original transcript to make sure that they were still a coherent interpretation. The clustered themes were given titles that reflected the connection between them, forming the superordinate themes of the analysis. At every stage, IS discussed the notes and themes with KK to reconcile any discrepancies. In the write-up of the analysis, relevant excerpts are presented alongside their corresponding subthemes.

### 2.6 Researchers' characteristics, positionality, and reflexivity

Both authors are female, middle-class, of South Indian descent, and familiar with the local Telugu language. The primary researcher (IS) is a medical sociologist and public health researcher with prior experience in coordinating clinical trials and training in Interpretative Phenomenological Analysis (IPA). Her familiarity with the clinical trial landscape may have influenced her reflections during data collection and analysis. However, as an outsider to the rural setting, she employed reflexive strategies, including engaging with critical feedback from the second author and bracketing assumptions, to minimize bias and ensuring the authenticity of participants' narratives. The second author (KK), a behavioural psychologist, public health academician, and bilingual researcher fluent in both Telugu and English provided supervisory oversight, ensuring methodological rigor, and offering a broader psychological and public health perspective to strengthen the study's interpretation.

### 2.7 Data trustworthiness

This study followed Lincoln and Guba's criteria for trustworthiness [30]. Prolonged engagement, triangulation, audit trail, and reflexivity were employed to ensure credibility, dependability, and confirmability. Prolonged engagement by spending sufficient time in the field allowed IS to develop rapport and build trust with the participants, understand the social context, and facilitate open, candid discussions for deeper insights. The participant quotations are literal translations from Telugu to English. The translated expressions were verified with the village representative. Following recommendations in cross-language qualitative research [31,32], minimal grammatical adjustments were made solely to aid readability, retain meaning and authenticity. IS and KK discussed interview content to triangulate interpretations, ensuring a shared understanding of participants' narratives. To establish dependability and confirmability, detailed field notes were maintained for all interviews, serving as an audit trail to document the research process.

## 3 Results

### 3.1 Participant characteristics

This study brings out the personal narratives of three ex-volunteers who discontinued clinical trial participation and three key informants of deceased serial clinical trial volunteers, all from remote villages from a mandal in Telengana, South India. All six participants belonged to the same mandal. Table 1, Table 2 present the basic characteristics of the ex-volunteers and key informants, respectively.

**Table 1:** Basic demographic details of ex-volunteers.

Ex-volunteer (EV*)	Age	Marital Status	Education	Present Occupation	Occupation before and during CT participation	Number of trials participated	Start-End Year	Total duration of participation	Currently participating	Alive/ Deceased	First source of information about CT	Relationship with other participants in the study
EV1	25	Single	High school completed	Employed in a private company	Newspaper seller	28	Mid 2015–2018	3.5 years	No	Alive, Ex-volunteer	From EV2 and friends	Neighbour of EV2, and living in the same mandal
EV2	32	Single	High school completed	Self-employed	Serving boy in catering company	20–25	2004–2016	12 years	No	Alive, Ex-volunteer	From EV3 and friends	Neighbour of EV1, and living in the same mandal
EV3	42	Married	Primary	Vendor	Vendor	19	2010–2018	8 years	No	Alive, Ex-volunteer (agent)	From friends	Living in the same mandal
EV4	53	Married	Primary	NA	Cook	9	NK	NK	NA	Deceased	Colleague at workplace	Living in the same mandal
EV5	44	Married	Primary	NA	Farmer	Over 10	2007 – NK	Over 5 years	NA	Deceased	From agent	Living in the same mandal

Note: EV*, Ex-volunteer, all EVs were male; NA, Not applicable; NK, Not known; CT, Clinical trials.

**Table 2:** Basic demographic details of key informant.

Key Informant (KI)	Gender	Relationship with ex-volunteer	Age of KI	Marital status	Education	Occupation
KI1	Female	Wife of EV4	36	Married	Primary	Housemaid
KI2	Male	Brother of EV5	50	Married	Primary	Farmer
KI3	Female	Mother of EV1	48	Widowed	Primary	Fruit vendor

Note: KI, Key informant.

All ex-volunteers were male, with an average age of 39 years. Both the deceased ex-volunteers were married and had children. Regarding education, two ex-volunteers completed high school, while the others did not progress beyond primary schooling. After exiting clinical trials, the three ex-volunteers secured livelihood through self-employment or salaried jobs. The number of trials each participant attended and the total duration of trial participation, as presented in the Table 1, are approximate figures provided by the participants, as they neither maintained definitive records nor could recall the exact details. The intervals between trial participation could not be determined, as participants admitted to not following the mandatory protocol of maintaining a gap between trials. The participants' initial source of information about clinical trial participation was from their next of kin, neighbour, friend, or colleague residing within the same mandal.

Three superordinate themes were generated from the IPA analysis, supported by a set of subthemes providing corresponding quotations to illustrate and substantiate them. Each quotation is identified with its respective participant number. A summary table of superordinate themes and subthemes is provided in Table 3.

**Table 3:** List of superordinate themes and subthemes.

Superordinate themes	Subthemes
1. A journey of strain and stain	1.1. Below the breadline: Coerced circumstances to over-volunteer 1.2. Labelled identity as ‘serial volunteer’ - a lifelong stamp 1.3. Being ‘stigmatised’ - a cultural taboo to battle 1.4. Physical and mental health succumbed to deterioration 1.5. Initial reliance on alternative beliefs and traditional practices for treatment
2. Transcending all turmoil to remain acceptable to system and society	2.1. Faking eligibility to remain compliant with trial entry 2.2. Consenting stands secondary to compensation negotiation 2.3. Lying to hide whereabouts 2.4. The joy of receiving pay cheques outweigh risk-taking 2.5. Enticing others to partake in the alternate profession
3. Agony of the family	3.1. Naïve about clinical trials 3.2. Families kept in the dark 3.3. Unhappy surprises - first confession about trial participation 3.4. Angry with the system 3.5. Practice continues

## 4 A journey of strain and stain

This superordinate theme sheds light on the burden borne by these individuals as they traverse a path fraught with strain (reflects the physical exhaustion, psychological stress, and economic precarity) and the indelible stains (signifies social and moral labelling) of their self-made choices, shaped by societal norms and structural inequities. The subsequent subthemes that follow sets the stage for a deeper exploration of their lived experiences.

### 4.1 Below the breadline: Coerced circumstances to over-volunteer

It is the dire need for money that coerced the ex-volunteers to partake in multiple trials. The decision to enrol was not driven by curiosity about science, but by the urgent need to provide for families living below the breadline. As KI1, the wife of a deceased EV4 who was on a contract job described how relentless financial strain shaped her husband's decision to participate. Her story reflects the burden of sustaining a household with limited income and dependents requiring care.

“*Our entire monthly family income is only 8000 rupees. I have a mentally disabled son. He doesn't know anything. He doesn't know to count cash and I'm afraid he gets cheated if I send him to house construction or labour work. Another son is a shop assistant and he wants to go to college. I don't earn much. It was the money problem. Actually, that was the problem. Actually, his father went to earn money from such trials*.”

For others, the motivation stemmed from their relentless struggles to meet the daily survival needs which forced the deceased EV5, who was a farmer, to rely on the financial benefits of clinical trials. As KI2, the brother of deceased EV5 shared,

“*My brother tried these trials because of reasons: big family, small income, loans, very difficult to survive. So he searched for other routes to earn money*.”

The prospect of easy money was tempting even when the risks were known. EV3 who had no savings confessed,

“*It was for some fever drug and I did not get much problems. I was tempted to do again when I needed money*.”

They were lured by easy access to information through agents (middlemen or brokers), messaging apps, and network groups, who often informally portrayed participation as low-risk and lucrative. As EV2 recalled,

“*Brokers tell, ‘You only have to come once, stay for 2 or 3 days, give a blood sample, you will get ₹20,000.*”

For some, repeated participation became normalized, a shift from need to dependency. EV1 reports engaging in 28 trials in a span of 2.5 years, disregarding study restrictions and ignoring health risks to maximize his earning potential. EV1 candidly admitted,

“*I have participated in at least 28 trials since 2015. Many of my friends were already doing it, so I gave it a try.*”

What began as a desperate bid for survival soon turned into a defining label, affecting their self-worth and shaping how the volunteers were viewed within their families and communities.

### 4.2 Labelled identity as ‘serial volunteer’ - a lifelong stamp

While the purpose of reliance on trials was for financial sustenance, it had a whiplash effect on the ex-volunteers, subjecting them to societal judgement, exclusion, and a lasting sense of alienation that extended well beyond their time as trial participants, leaving a negative connotation, an internally constructed identity by the ex-volunteers themselves. Although none of the ex-volunteers or their families used the exact English phrase “serial volunteer”, teasing remarks often uttered in jest carried similar undertones. EV1 described how this invisible tag shadowed him long after he stopped participating,

“*They remind me of my past even if I want to move on*.” They say, “*You made money through those short-cuts. Why complain now?*”

EV3 internalised self-stigma and explains his account of public judgement,

“*People looked at me differently once they found out that I participated in trials. They stopped trusting me.*”

Similarly, EV2 confessed to the isolating shame that came with this label, “*I feel ashamed when going out. People were not ready to talk to me after knowing this*.”

For some, interactions with non-staff outside the lab exacerbated feelings of dehumanization, as they felt being treated more as “data points” than like real people. EV2 shared,

“*Earlier, there were few foreigners who came to visit me with a doctor. They interviewed me and left*.”

Such encounters contribute to a sense of objectification, where the ex-volunteers question themselves “Is my worth reduced to a utility as an experimental subject?”

### 4.3 Being ‘stigmatised’ - a cultural taboo to battle

As the news spread and the neighbourhood become aware of their repeated involvement, the stigma becomes particularly pronounced, often leading to subtle forms of exclusion or pity. This stigma forced the ex-volunteers to navigate a double burden dilemma – enduring the physical and emotional toll of trials while battling the societal norms that ostracize them.

In the conservative cultural context of rural South India, the human body is often viewed as sacred and using the body for financial gain provoked gossip and suspicion. EV1 captured this moral framing vividly,

“*Subjecting our body to multiple pricks and medications is cruel and an act against God's will. We are looked at with suspicious eyes.*”

Secrecy and stigma coexisted but at different social levels and time points. During participation, most ex-volunteers concealed the truth due to anticipated stigma and from fear of being outcast due to the cultural taboo, which further compounds their vulnerabilities and leaves them without a support system. EV2 explained how he chose silence over disclosure fearing ridicule,

“*No. They hesitated to talk to me. Family shows sympathy*. *I had no choice but to keep it a secret. Society does not understand what we go through. They judge us*.”

The ex-volunteers internalize these negative perceptions, which causes them to self-doubt and feel guilty. EV3's reflection illustrates this painful duality,

“*I feel like I have done something wrong. Even though I earned money for my family, people look at me like I am worthless*. *It's a constant struggle. I know why I did it, but others don't care. They only see the shame*.”

Alongside internalizing stigma, the repeated strain of participation soon began to surface on their physical and mental health.

### 4.4 Physical and mental health succumbed to deterioration

Over time, the ex-volunteers put themselves at risk through their journey of participation without adequate recovery periods. For many, the consequences were irreversible. EV2 who had participated in over twenty trials reflected on the decline in his overall health,

“*Because of these trials, I can't live like I used to. I am no longer healthy*.”

EV2 shared the breadth of his physical ailments,

“*Chest pain, nerve problems, giddiness in the head because of which I used to get severe pain at the back of my head, knee pain. I cannot eat when I have these pains*.”

Likewise, EV3 who has participated in over nineteen trials, acknowledged lingering health effects that impeded his ability to work, “*After my previous participation in trials, my body is not fine to do my job properly*.”

Similar experience was shared by EV1, a deterioration of his physical condition following prolonged participation (about twenty-eight trials),

“*It was not good. Because I had all these tablets, my body affected. Bones, chest, skin problem. Health is gone*.”

The combination of physical strain, hasty travelling, long waiting times during screening, lengthy stays at the labs and hostels, drug side effects, and the psychological burden of their participation led to symptoms such as paranoia, anxiety, auditory hallucinations, and confusion. EV1 described a particularly distressing episode after a mental health study,

“*In the last study, mental study, I completed four periods. It became too much for me. When I was coming in train, my head was becoming disturbed. I started hearing voices. I felt like someone was talking to me. When I turned around, there was nobody. I thought it's a problem. It might be headache only*.”

The paradoxical situation where participation temporarily alleviates poverty, and simultaneously erodes health and resilience, presents blurred boundaries, leaving the individuals more vulnerable than before.

### 4.5 Initial reliance on alternative beliefs and traditional practices for treatment

The families believed that the unusual behaviours or symptoms exhibited by the volunteer's post-trials were caused by spiritual imbalances or supernatural forces. Resorting to traditional and spiritual practices was the first line of healing before any medical intervention was considered. EV1 recounted,

“*First when I was there, I didn't feel anything. Only when returning in train, I started hearing voices. At home also, I heard voices. My mom rubbed lemon on my head. When this was not helping, they took me to temple. The Bhabha temple, Dharga. After going to temple, my health problem worsened*.”

The use of lemons, a symbol of spiritual purification, is a common practice to ward off evil influences in most Indian shouseholds. This narrative by EV1's mother KI3 describes the ordeal of seeking spiritual remedies,

“*I took him to bigger Dharga. He started rolling on the floor and became restless. We believed he was demon-possessed. There used to be horses in the Dharga. He started jumping on them. He used to go to graveyard besides the Dharga and say, ‘there are demons here. I am also going to die’. I have bought kilograms of lemons for him. These lemons will be squeezed, without sugar, salt or water. This was given to him to drink. He used to spit away everything. We tied up his hands and legs. I took him to Bhabha house thrice, nothing happened. I gave up*.”

Despite these expenses and efforts, the symptoms persisted or worsened, leaving his family in despair. The family questioned their continued failure of traditional practices, eventually, resorting to formal medical help or identifying links to clinical trial participation to seek alternative solutions. EV1's mother shared, “*For two months, I struggled, taking him to different Dhargas. Then we got the ID card of the lab*.” In EV1's case, his mother approached the laboratory for compensation, and protested outside their home, hoping to draw attention from the media, politicians, and the public. However, due to the sensitivity and legal implications surrounding the issue, little information was revealed to the researcher. Eventually, EV1 sought treatment at a government hospital. To meet the costs of medications and travel, his mother borrowed money from acquaintances and charitable institutions.

Likewise, EV2 recalled using home remedies to manage side-effects, “*When I get headache, I prepare kashayam*” (EV2). ‘*Kashayam*’ is a traditional herbal concoction made from various herbs and spices, commonly used to promote respiratory health and overall physical wellness.

## 5 Transcending all turmoil to remain acceptable to system and society

This superordinate theme and the subsequent subthemes explore the delicate balancing act of ex-volunteers to remain compliant with the clinical trial system while simultaneously navigating the social and personal challenges that accompany their participation.

### 5.1 Faking eligibility to remain compliant with trial entry

Deceit for profit was used as a survival tactic. It is a precarious balance these individuals maintain by bending the rules to navigate the rigid inclusion criteria of clinical trials. Altering age in enrolment forms, or disclosing incorrect date of birth was done to fit the study requirements. For instance, from the narrative of the wife of the deceased EV4, it was known that despite being over 50 years old, the submitted identity documents claimed he was 39 to qualify for a study restricted to participants under 45 years of age. The deceit was exposed only after his death was reported in the media.

Another common tactic by the ex-volunteers was to misrepresent their medical history to avoid disqualification, such as hiding pre-existing health conditions, denying use of other concomitant medications, non-disclosure of alcohol intake or smoking habits. EV1 who had recurring headaches and mild hypertension from previous trial exposures admitted to hiding his condition during screening,

“*It is common for people to tell lies. I said, I do not take any medications, I do not drink and smoke. I pass the test*.”

Some ex-volunteers even carry supplements like iron, folic-acid, and multi-vitamins to mask conditions like anaemia before screening. EV1 shared his experience of taking over-the-counter supplements,

“*No problem even if the blood content is low. I bought iron tablets from the pharmacy and took it for few weeks before screening*.”

This tendency to manipulate eligibility was mirrored in their approach to consent.

### 5.2 Consenting stands secondary to compensation negotiation

Bargaining over compensation, time duration, and accommodation takes precedence over understanding the risks involved and trial procedures, reducing the consent process to a negotiation of terms rather than an informed decision. The “negotiation” does not imply formal bargaining power but rather comparing rates between study sites or discussing expectations with brokers and friends before arrival. The narratives reveal that ex-volunteers come mentally prepared to participate in the trial well before the consent process, rendering the session a mere formality.

“*I had already decided to participate before they explained anything. The session was just routine for me. I was more concerned about the payment*,” admitted EV3.

The ex-volunteers had been signing the dense and lengthy informed consent forms without reading or fully understanding its content.

“*The last study was for a mental tablet. They explained the consent form but I did not read it properly. I could not read the full details*,” EV2 admitted.

The pressure to complete the formalities quickly leaves them with lack of ability or opportunity to ask critical questions, undermining the mutual consenting process. EV2 noted,

“*I signed and dated all the 46 pages in one hour. It was too much to read or understand*.”

Sometimes, the ex-volunteers receive verbal assurances rather than a copy of the informed consent form, not knowing they deserve to have a copy, leaving them unaware of the study's risks or implications. EV2 shared,

“*They said there was 106 side-effects. But they assured us that such experiments are already on many people, these drugs are available in the market, and that we should not worry. I then signed the form*.”

The same personal and financial pressures that overshadowed consenting also compelled volunteers to conceal trial involvement through fabricates stories.

### 5.3 Lying to hide whereabouts

To overcome internal conflict, secrecy became an act of self-preservation, an emotional and social coping mechanism to manage stigma within their communities and maintain acceptance within the family. As participation in trials is considered a morally questionable act, fabricating alternative narratives to conceal true whereabout becomes a necessary strategy to avoid scrutiny from family members. EV1 shared how he disguised his involvement by claiming employment elsewhere, “*I would say I have a week's job in the city*.”

Similarly, the wife of the deceased EV4 noted, “*He used to say, he is going for catering, a hotel job*.” Such statements reveal that participants constructed believable narratives aligned with socially acceptable occupations.

These false claims allowed the ex-volunteers to leave their homes unnoticed, deflect questions, avoid judgement, and keep their participation in trials hidden from others.

Their absence eventually raised suspicions later, affecting the relationship between the ex-volunteers and the family members. The brother of the deceased EV5 recalled his frustration upon learning the truth,

“*I used to be working. When I called, he used to lie telling, ‘There is no signal. Will call you later.’ Had he informed me about these trials, I would not have let him go*.”

These accounts suggest that deception was not motivated by guilt alone but by the perceived need to shield their families from worry, shame, or scrutiny. The acts of concealment driven by necessity transformed into a calculated pursuit of pay cheques that outweighed concerns for health or risk.

### 5.4 The joy of receiving pay cheques outweigh risk-taking

The substantial pay cheques or on-the-spot cash from serial volunteering is a dominant motivator and a dangerous incentive that eclipses trial safety protocols and health risks. Payments ranging from ₹20,000 to ₹25,000 for a week-long commitment far exceed what a daily wage worker might earn in the same period. This disparity transforms trial participation into a perceived opportunity rather than an ethical dilemma. EV2 noted how he outweighed physical discomfort and fear in exchange for monetary reward,

“*I considered dropping out of one study due to severity of side effects. Later, I changed my mind because it had good payment. I was more careful about choosing trials after that*.”

Similarly, EV1 endured body pain, fever, and headache during a trial but stayed until the end to secure the lump-sum payment ₹24,000, as he was able to pay off his debts,

“*By the last period, many had dropped out but I stayed because I needed the money. I completed the study and it was worth it*.”

Eagerness to enrol in high-paying studies drives the ex-volunteers to contact the agents offering lucrative opportunities, despite disregarding rules or protocols. EV1 candidly admitted, the search for the next high-paying trial often began immediately after completing one,

“*I ask, ‘Anna, is there any new study.’ The agents will say, ‘Yes. There is a study for ₹10,000 to ₹15,000 at so and so lab. Wherever the payment is high, I go there*.”

The perceived profitability, coupled with contact with agents, prompted the current volunteers to persuade others in their community to join clinical trials.

### 5.5 Enticing others to partake in the alternate profession

Participation in clinical trials gradually evolved into what participants described as an “alternate profession”. The primary way newcomers knew about clinical trials was through word-of-mouth from persons who have participated before. EV1 recounted,

“*EV2 and I were neighbours. We grew up together. I was asking for some money, and he mentioned about clinical trials. He explained that they give medicine, take blood samples, pay ₹13,000 to ₹15,000, must stay for 2-3 days*.”

With increasing smartphone access, messaging Apps becomes a potent recruiting tool, circulating updates about ongoing studies and varying compensation rates across centers and states. EV2 explained,

“*First time when I went to the city, my friends were talking about this. Through them, I came to know about clinical trials through an App. They said payments were less in one center but much higher in another state, up to ₹20,000 or ₹25,000*.”

Such communication networks fostered a sense of belonging among like-minded individuals. However, their families who disapproved their choices, quietly endured the resulting distress and misunderstanding.

## 6 Agony of the family

While participants initially concealed their involvement in clinical trials to avoid anticipated stigma and social judgement, their secrecy could not be sustained indefinitely. This superordinate theme unearths the family's experience of shock, confusion, and anger as they deal with the eventual revelation, either an illness or aftermath of their family member's trial participation, uncovering hidden evidence, navigating tragic consequences, and confronting systemic failures.

### 6.1 Naïve about clinical trials

Neither the ex-volunteers nor their families are fully aware about clinical trials. They have only vaguely heard about it, often through rumours or media reports of unfortunate incidents. The ex-volunteers neither comprehended the written information on the consent form, nor asked questions to the research staff. They made no efforts to consult with their trusted doctor or someone in their family. The wife of the deceased EV4, still grappling with the shock of discovering her husband's hidden participation recalled with quiet regret,

“*Had he mentioned or shared the documents with me, I would have learnt about it*. *I wish he had mentioned about what was going on in his mind and body.*”

A deeper issue is the widespread lack of awareness due to inadequate communication and education about clinical trials in a locality that houses many CROs. The brother of the deceased EV5 explained voicing a widely held misconception,

“*Actually, we do not know that clinical trials will happen this way. Specially, we did not know they will do on humans. We believe that they will do trials on animals like rats*.”

### 6.2 Families kept in the dark

The families are left unaware of the source of income that sustains them. The wife of the deceased EV4 believed that her husband, a primary breadwinner, earned his living as a cook at weddings, travelling frequently for catering assignments, reflected,

“*We thought he was travelling around with catering companies. This was a shock to us*.”

Tragically, only after his death, she realised that the income was attributed to trial participation. Such revelations can be devastating.

To keep their involvement hidden, ex-volunteers went to great lengths to destroy any trace of involvement in trials. They actively destroyed evidence documents such as insurance papers, consent forms, and study participation cards. EV1 admitted,

“*I will receive it from labs but will not bring them home. I will tear them. I did not want my family to know. I was scared there will be problem if they came to know*.”

The secrecy is compounded by the lack of preparedness for emergencies. The brother of the deceased EV5 sobbingly expressed how he felt deceived and helpless by his brother for having chosen secrecy over trust,

“*He should have told us, ‘I am not well from taking part in clinical trials.’ Had he said this, I would have asked others for help*.”

### 6.3 Unhappy surprises - first confession about trial participation

Despite their efforts to keep the truth hidden, the secret of trial participation inevitably surfaced, often through chance discoveries or unusual behaviour that could no longer be ignored. At one instance, the truth was uncovered while sorting through the ex-volunteer's possessions, where parts of consent form and study participation card was tucked away underneath clothes in the cupboard. EV2 described how the revelation unfolded unexpectedly,

“*My sister was clearing things in the shelf. That time, she found an ID card from my bag that belonged to a lab*.”

At another instance, the discovery occurred when EVI was caught attempting to destroy evidence. He recalled how his mother confronted him,

“*First time, I remembered one day my mom caught me tearing some papers. Second time, when she was washing my clothes, she noticed some papers and asked me, ‘Your signatures are there in these papers.’ I lied, saying these are office papers. Only after finding these evidences, they came to know I was taking part in clinical trials. I was taken to the hospital later*.”

Sometimes, it was not paperwork but physical and behavioural changes that exposed the secret. The unsettling behaviour led family of EV1 to suspect something was wrong.

“*How we came to know is: He started talking absurd. We normally wear one dress and one pant. He used to wear six shirts and six pants at a time. He used to say, ‘I am demon-possessed.’ He used to say, ‘I'm hearing voices through the walls*.”

These unhappy surprises from their loved ones transformed into strained relationships with the family, setting the stage for enacted stigma, grief, and systemic anger that followed.

### 6.4 Angry with the system

For the affected families, anger was the only response left after repeated attempts to seek help or justice met with silence. They expressed frustration for the lack of transparency and accountability from the trial centers, and agents particularly during times of medical and financial crisis. When volunteers fell ill or experienced severe side effects, the burden of care, hospitalisation, travel, and medication costs fell entirely on the families. EV3 expressed this resentment with rage,

“*They will use us, but we will have to pay for all the expenses. Neither the company nor the agent will pay for the expenses*.”

The role of agents, middlemen, or brokers, who acted as intermediaries between volunteers and trial centers also came under heavy criticism. They disappear when complications arose. The grieving wife of EV4 lashed out saying,

*“The agent is behind money. He is not bothered about anything else*. He ignores our contact.”

The sense of betrayal was so deep that the volunteers refused further communication with anyone linked to the trial system, including the agents. EV3 said,

“*Those who do not know, will call. If call comes, I tell them about the problems I faced and disconnect. If an agent calls, I tell, ‘because of you, I got this problem’ and then disconnect.*”

This frustration, however, soon turned into resignation, as the ex-volunteers and their families recognised that despite their personal losses, their protests and voices alone could not change a system too vast and that the practice of serial volunteering persisted unabated.

### 6.5 Practice continues

Despite the risks and ethical concerns, clinical trial participation is an ongoing reality in India for those facing systemic issues, such as poverty, limited job opportunities, and marginalized living. For many, the decision to stop enrolling was not born from empowerment, but from physical, emotional, and moral exhaustion. We can recall from the accounts of ex-volunteers in the previous subthemes, how deteriorating health and family distress eventually forced them to withdraw. Their bodies could no longer endure repeated blood draws, confinement, and side effects; nor could their families bear the anxiety of watching them suffer. However, stories of victims who gave up serial volunteering due to adverse experience or increased visibility does not deter the cycle of new recruits.

“*This industry is attractive*” in the words of EV3.

The lure of instant cash continues to draw new entrants, including students and unemployed youth. These young individuals prioritize short-term monetary benefits over the long-term health risks involved. As EV1 observed,

“*Yes, there is money problem. There are many who are still doing it*.”

EV1 explained how these networks operate.

“*Students, college degree students from different places come. I have personally seen many. They say they have project work and go. Some will say, they have one-week job and go to a different place for clinical trials*”.

For them, participation is a promise of quick financial gain when other opportunities are scarce.

The voice of former volunteers goes unheard in the face of economic desperation. EV2 advised,

“*They should not over volunteer in such trials and spoil their health. My suggestion is nobody should go for back-to-back trials*.”

Collectively, these experiences reveal an underground economy of trial volunteering, reminding us that the issue extends far beyond the lives of these few participants.

## 7 Discussion

The qualitative study captures the multifaceted challenges faced by the ex-serial clinical trial volunteers and their families, as they enter the world of clinical trials seeking financial relief, only to face a cascade of physical, emotional, and social consequences. Their lived experiences reflect a complex interplay of financial desperation, health deterioration, and societal stigma. The decision to participate in one trial after another, sometimes with gap or without gap, is compelled by their survival needs, driven by illiteracy, poverty, unstable income, lack of savings, mounting debts, and emergency family needs. The allure of on-the-spot pay cheques or ready cash, facilitated by agents, or peer networks, transformed clinical trials into a part-time job for many, with messaging apps further enticing others to join. Their narratives echo findings from Monahan and Fisher's qualitative entrepreneurial interviews from healthy volunteers across the U.S. where they rationalize these reasons for taking part in clinical trials [13], and Krishna and Prasad (2014), whose case study of a CRO in Hyderabad, India, highlighted financial desperation as the primary motivator for male breadwinners aged 18–46 years to participate in clinical trials [10]. Similarly, Doshi et al. (2013) identified financial incentives as the predominant motivating factor for healthy participants and patients to consent for non-therapeutic trials at a tertiary care centre in Mumbai, India [33]. Conceptually, Cooper and Waldby (2014) situate human subjects within biomedical economy as ‘clinical labour’ [34] and Monahan and Fisher (2020) describe as ‘sacrificial labour’ [35], where these ex-volunteers make temporary compromises of their bodies both as site and as means of economic endurance within the conditions of systemic inequalities and insecurity. For them, contribution of trials to science is either unknown or disregarded. The framing of clinical trials as income-supplementing opportunities, rather than scientific enterprises reflects this lived dissonance and underscores structural compulsion rather than agency.

Unlike studies from high-income contexts such as Abadie's (2010) The Professional Guinea Pig [22] and Fisher's (2009) Adverse Events [36], where serial volunteers in the U.S. often display informed awareness, community solidarity, and calculated risk-taking, the Indian volunteers' understanding of the broader purpose of research is restricted to superficial details, such as drawing blood or the possibility of mild side effects, as observed in earlier studies in India [[16], [17], [18], [19], [20],^37^]. The international literature [23,38] has shown that serial participation can sometimes foster “experiential expertise,” enabling participants to manage risks and negotiate study conditions more strategically. In contrast, volunteers in this study perpetuated dependence rather than expertise and do not accumulate ‘experiential capital’ as seen in the non-Indian contexts. Speaking of consent, it was not a genuine expression of understanding or agreement but rather a passive participation process in securing monetary reward. These explicit factors limit their capacity to comprehend the risks and benefits of the trial and constrain their autonomy to refuse participation. A systematic review by Johnson et al. (2017) [21] and the VolREthics initiative [40], a global effort to safeguard the well-being of healthy volunteers in biomedical research, underscored that informed consent must emphasize risk transparency and ensure comprehension, which is often overlooked in low-income settings. This study highlights the importance of public education to dispel misconceptions and promote informed decision-making, suggesting that targeted campaigns through NGOs or identified educated professionals within the affected community could bridge the knowledge gap. Finding ways to incorporate these measures will require imaginative thinking and culturally adaptive language.

Their fragmented knowledge about clinical trials is reflected in their risk-taking behaviour. The trial volunteers' resort to manipulating documents, falsifying personal details, masking health conditions, and ignore re-eligibility locking days, to meet the eligibility criteria. This subversive behaviour is exhibited to competitively secure a slot in subsequent trials. The studies by Dresser (2013) [41] and Monahan and Fisher (2015) [13] also noted similar deceiving behaviours. Some travel to different states to prevent investigators from linking their prior participation history. Edelblute and Fisher (2016) noted similar patterns in the U.S., where serial participants manipulated screening tests and travelled across states to maximize earnings [42]. To avoid scrutiny from family and society, the ex-volunteers prefer to conceal all evidence about their involvement in trials by hiding, burning, or tearing study participation cards and signed consent forms, or fabricate stories to justify their prolonged absences from home, all of which jeopardize the integrity of the trials and perhaps endanger their own health. These strategies help volunteers transcend life's turmoil, constructing a fragile yet persistent sense of acceptability within the system and their community.

These ex-volunteers voice out how they were teased at due to their involvement in multiple trials led to an enduring identity of being called “serial volunteers”, operating as a spoiled identity as ascribed by Goffman in his Classic Stigma theory [43] and becoming a source of inner conflict, a subject of societal judgement and seclusion within their communities. Scholars who studied the repeated participation in the context of healthy volunteer research call this distinct occupation identity, the “professional guinea pig” which carries negative connotations [22]. In India's collectivist cultural context, where reputation and family honour are deeply valued, clinical trial participation is often shrouded in secrecy due to the cultural taboos, against divine will, and societal stigma surrounding such activities. This stigma leads to diminished social interactions, creating an invisible barrier between them and the others they know. The cultural taboo perpetuates systemic inequality by hampering open discussions about their needs and difficulties. Analytically, recognising the feedback loop process as a result of the stigma: social exclusion, secrecy, and marginalization help explain why volunteers hide trial involvement from families, why families learn only after adverse events, and why the label endures long after participation stops.

Families of serial volunteers who are left in the dark, bear the brunt of this secrecy and wilful deception. Stories of neighbourhood residents who suffered side-effects or lost their lives from covertly participating in a string of clinical trials fostered fear and apprehension, as confirmed from the findings of a community survey conducted in the village where the news of deceased volunteers surfaced in Telengana, India [17]. Families are left betrayed after learning of the volunteers' involvement in trials, often indirectly or after unfavourable things have happened. They are left unprepared to support their loved ones in navigating the risks and responsibilities of trial participation. This finding echoes the expropriation experiences of trial volunteers from Mumbai and Hyderabad in India by Wadkar (2021), contributing to normalization of risks, where repeated participation of volunteers in the current study desensitizes them to danger, reframing risk-taking as routine livelihood practice rather than health concern [16].

The cumulative effect of repeated drug exposures, noncompliance with trial procedures, and disregard for washout recovery periods leaves volunteers with debilitating health issues and psychological anguish. However, the risks described by participants in this study appear far greater than those reported in international literature. Johnson et al. (2017) noted that most Phase I trials globally have low incidences of severe harm [39]. For the volunteers in this study who already living at the margins, even temporary side effects such as dizziness, fatigue, or headaches can disrupt livelihood, reinforcing the perception that their health has been permanently compromised. This discrepancy may not necessarily reflect the objective biomedical risk, but rather how participants make sense of their experiences through embodied suffering, regret, and moral dissonance.

In the aftermath of adverse physical and mental health effects from clinical trial participation, the volunteers, and their families' first choice of help-seeking was spiritual healers and traditional medicine, reflecting the cultural underpinnings of their coping mechanisms. The transition to seek medical help often comes after the families have incurred significant financial, emotional, and physical loss highlighting the gap between conventional practices and modern healthcare systems, emphasizing the need for structural interventions, such as post-trial healthcare access and fair compensation, as suggested by Wadkar (2021) [16], to address the long-term health and social consequences of trial participation.

Krishna and Prasad (2014) highlighted the absence of a centralized registry in India, enabling trial volunteers to engage in consecutive trials undetected [7]. Establishing such registries, as suggested by Urooj et al. (2017) [20], Bompart 2019 [24], and Foster & Malik (2012) [45], and as recommended by VolREthics Initiative [40,44] could mitigate these practices by tracking participation and ensuring compliance with washout protocols, thus enhancing safety and data integrity. The Over-Volunteering Prevention System (TOPS), an internet-based system run by the UK Health Research Authority is an example to cite as an installation measure to facilitate tracking and monitor repeat volunteering [46].

## 8 Limitations and future research

This study has several limitations that must be acknowledged. First, the research is confined to a rural setting, making it difficult to replicate the findings in other contexts, such as urban or semi-urban environments, where the dynamics of clinical trial participation may differ. Second, the limited number of participants, which, while suitable for an IPA study, and the purposive sampling approach limits the study's generalizability and scope, as the responses are specific to the personal experiences of ex-serial volunteers and key informants, reflecting their unique interactions with clinical trials. Additionally, the study does not include perspectives of currently active clinical trial volunteers, agents, laboratory, or CRO representatives which could provide a more comprehensive understanding of the system. Future research with a larger sample and relevant stakeholder participation may provide additional perspectives on serial clinical trial volunteering.

This study captures participants' immediate and retrospective experiences rather than long-term outcomes. Prospective qualitative or mixed-methods designs could explore the retention of knowledge, shifts in perceptions, and evolving physical and psychological consequences of serial trial participation over time. This study's reliance on participants' memories and recollections further poses the risk of recall bias, particularly given the extended timeframe over which their experiences occurred. Subtle nuances may have been lost in the translation of the interviews, and the nature of qualitative research also introduces inherent subjectivity, as the data obtained may be interpreted differently by various readers. Despite these limitations, the study provides valuable insights into a hard-to-reach population, offering a nuanced understanding of the lived experiences of ex-serial clinical trial volunteers and their families. The findings have implications for ethical oversight, policy updates, and participant protection globally. Future research in early-phase and bioequivalence trials should explore these experiences across diverse geographic and socio-economic settings across low- and middle-income regions, to evaluate broader transferability of the findings and inform equitable and sustainable clinical trial practices.

By amplifying the voices and vulnerabilities of underserved and underrepresented individuals engaged in drug development processes, this work aligns with the World Health Organization's (WHO) global commitment to streamlining oversight [47] and strengthening the clinical trial ecosystem worldwide [48]. Specifically, it contributes to the objectives outlined under Sustainable Development Goal (SDG) 3: Good Health and Well-being [49], including Target 3.8 on achieving universal access to safe, effective, and quality health services, Target 3.b which supports research and development of affordable medicines, vaccines, and equitable access to health innovations and SDG 10: Reduced Inequalities [49].

## 9 Conclusion

This study provides a rare qualitative insight into the lived experiences of serial clinical trial volunteers in rural South India, revealing how social marginalization, economic instability, financial desperation, and limited awareness trap vulnerable individuals in a cycle of exploitation. For many, participation in clinical trials becomes a means of livelihood rather than an informed or voluntary choice. The cascading consequences of physical, emotional, and social harm underscore the systemic gaps in ethical oversight, participant education, and post-trial protection. By documenting voices that are often unheard, this study extends existing literature by adding an understanding of the socio-cultural and emotional realities surrounding translational serial volunteering. Despite existing government regulations, the underground nature of recruitment networks and the persistent demand for willing participants enable these practices to thrive. These findings call for the establishment of a national registry to track participation, alongside strengthened policies and enforcement mechanisms to ensure transparency, ethical recruitment, and the safety of vulnerable populations. Addressing these systemic challenges is critical to making clinical research in India both scientifically sound and socially responsible.

## Funding

This research did not receive any specific grant from funding agencies in the public, commercial, or not-for-profit sectors.

## Ethics approval

The study was approved by the Institutional Review Board of SRM School of Public Health, SRM Institute of Science & Technology (IRB/PH-25/2018).

## Availability of data and materials

No datasets were generated for this study. The findings are based on qualitative data, and the derived themes are presented alongside excerpts from participants' verbatims to support the interpretations. The raw data (interview notes) are not publicly available to ensure participant confidentiality.

## CRediT authorship contribution statement

**Irene Sambath:** Writing – original draft, Visualization, Validation, Methodology, Investigation, Formal analysis, Data curation, Conceptualization. **Kalpana B. Kosalram:** Writing – review & editing, Visualization, Supervision, Methodology, Data curation.

## Declaration of competing interest

The authors declare that they have no known competing financial interests or personal relationships that could have appeared to influence the work reported in this paper.

## Appendix A Supplementary data

Supplementary material Image 1
