# Supplementary material for: “Stories like ours continue”: A phenomenological exploration of the lived experiences of ex-serial clinical trial volunteers in South India
Source: Dialogues Health. 2025 Nov 7;7:100256. doi: 10.1016/j.dialog.2025.100256 (PMC12648981; doi:10.1016/j.dialog.2025.100256)
Supplement: Supplementary file 1 — Supplementary material [file mmc1.docx]

# **Supplementary Material S1**

**In-Depth Interview Guide for persons who have taken part in multiple clinical trials (in English)**

Thank you for agreeing to participate. I want to assure you that I am not a doctor and have no connection with pharmaceutical companies or health authorities. This study aims to understand the experiences of individuals who have participated in multiple clinical trials and the impact it has had on their lives and families. There are no right or wrong answers – I am here to listen to your story.

Your responses will remain strictly confidential, and your name will never be revealed. You may stop the interview at any time.

Can you tell me a bit about yourself? (Age, education, occupation, family background)

How long you have been living in this village? What does this community mean to you?

How did you first hear about clinical trial participation? (Probe: Friend, WhatsApp group, mobile text messages, agent, pharma company advert, doctor, others?)

For how long have you been participating in clinical trials?

Are you still taking part in trials? (If stopped, why did you stop?)

How many times have you participated in clinical trials?

What were the reasons for participating in multiple trials? (Probe: Financial need, joblessness, debts, family emergency, peer influence, others?)

Did you discuss about your decision to take part in trials with anyone you knew? (Probe: Spouse, parent, sibling, children, friend, doctor?)

How did your family react when they found out about your participation?

How did your participation affect your relationships with family and the community?

Can you describe a typical day during a clinical trial? (Probe: What were the procedures? What were you asked to do?)

What was your understanding of the risks before participation?

How was the informed consent process conducted? Did you feel you fully understood the details?

Did you adhere to the required washout periods between trials? If not, what influenced your decision? (Probe: Financial reasons, agent influence, peer pressure?)

Have you experienced any short-term or long-term health problems during or after participating in trials? (Probe: side effects) How did you cope with these health issues?

Did you ever feel mentally or emotionally distressed during or after participating in trials? How did you cope with these feelings?

Were you able to seek treatment?

Did you receive any compensation? How did the payments from clinical trials affect your daily life? (Probe: Was it enough? Met basic survival needs?)

From your overall experience, how did it feel to be a volunteer for such clinical trials?

Can you think of any good things that have happened to you because of your participation? (Probe: What was it like? How did you feel? Did it improve your life in any way?)

Can you think of any bad things that have happened to you because of your participation? (Probe: Why was it bad? How did you cope?)

Do you think people from your community will continue participating in trials despite the risks? Why or why not?

Is there anything else you would like to share about your experience?

How have you found this interview?

Thank you for sharing your experiences with me. Your insights are valuable and will help in understanding the realities of serial clinical trial participation.

**In-Depth Interview Guide for persons who have taken part in multiple clinical trials (in Telugu)**

**బహుళ క్లినికల్ ట్రయల్స్‌లో పాల్గొన్న వ్యక్తుల కొరకు సవివరమైన ఇంటర్వ్యూ గైడ్ (తెలుగులో)**

ఈ కార్యక్రమంలో పాల్గొనడానికి అంగీకరించినందుకు ధన్యవాదాలు. నేను వైద్యుడిని కాదని, నాకు ఫార్మాస్యూటికల్ కంపెనీలు లేదా ఆరోగ్య అధికారులతో ఎటువంటి సంబంధం లేదని మీకు భరోసా ఇవ్వాలనుకుంటున్నాను. ఈ అధ్యయనం బహుళ క్లినికల్ ట్రయల్స్‌లో పాల్గొన్న వ్యక్తుల అనుభవాలను మరియు వారి జీవితాలు మరియు కుటుంబాల పై ఆ ట్రయల్స్‌ ప్రభావాన్ని తెలుసుకోవడం కొరకు ఏర్పాటు చేయబడింది. ఇందులో సరైన లేదా తప్పు సమాధానాలు లేవు - మీ కథ వినడానికి నేను ఇక్కడ ఉన్నాను.

మీ సమాధానాలు ఖచ్చితంగా గోప్యంగా ఉంటాయి మరియు మీ పేరు ఎప్పటికీ వెల్లడించబడదు. మీరు ఏ సమయంలోనైనా ఇంటర్వ్యూను ఆపివేయవచ్చు.

మీ గురించి కొన్ని వివరాలు చెప్పగలరా? (వయస్సు, చదువు, వృత్తి, కుటుంబ నేపథ్యం)

మీరు ఈ గ్రామంలో ఎంతకాలంగా నివసిస్తున్నారు? ఈ కమ్యూనిటీ మీకు ఎంత ముఖ్యం?

క్లినికల్ ట్రయల్‌లో పాల్గొనడం గురించి మీరు మొదట ఎలా విన్నారు? ( పరిశీలన: స్నేహితుడు, వాట్సప్ గ్రూప్, మొబైల్ టెక్స్ట్ మెసేజ్‌లు, ఏజెంట్, ఫార్మా కంపెనీ ప్రకటన, డాక్టర్, ఇతరులు?)

మీరు ఎంతకాలంగా క్లినికల్ ట్రయల్స్‌లో పాల్గొంటున్నారు?

మీరు ఇంకా ట్రయల్స్‌లో పాల్గొంటున్నారా? (ఆపివేస్తే, ఎందుకు ఆపారు?)

మీరు క్లినికల్ ట్రయల్స్‌లో ఎన్నిసార్లు పాల్గొన్నారు?

బహుళ ట్రయల్స్‌లో పాల్గొనడానికి కారణాలు ఏమిటి? (పరిశీలన: ఆర్థిక అవసరం, నిరుద్యోగం, అప్పులు, కుటుంబ అత్యవసర పరిస్థితి, తోటివారి ప్రభావం, ఇతరులు?)

ట్రయల్స్‌లో పాల్గొనాలనే మీ నిర్ణయం గురించి మీకు తెలిసినవారు ఎవరితోనైనా చర్చించారా? (పరిశీలన: జీవిత భాగస్వామి, తల్లిదండ్రులు, తోబుట్టువులు, పిల్లలు, స్నేహితుడు, వైద్యుడు?)

మీరు ట్రయల్‌లో పాల్గొనడం గురించి తెలుసుకున్నప్పుడు మీ కుటుంబం ఎలా స్పందించింది?

మీరు ట్రయల్‌లో పాల్గొనడం అనే అంశం మీ కుటుంబం మరియు సంఘంతో మీ సంబంధాలను ఎలా ప్రభావితం చేసింది?

ట్రయల్స్‌లో పాల్గొనడానికి ముందు ప్రమాదాల గురించి మీకు ఉన్న అవగాహన ఏమిటి?

క్లినికల్ ట్రయల్ సమయంలో సాధారణంగా ఒక రోజు ఎలా గడుస్తుందో మీరు వివరించగలరా? (పరిశీలన: విధివిధానాలు ఏమిటి? ఏం చేయమని అడిగారు?)

సమాచారం తెలిపిన తర్వాత సమ్మతి ప్రక్రియ ఏవిధంగా నిర్వహించబడింది? మీరు వివరాల గురించి పూర్తిగా అర్థం చేసుకున్నారని భావిస్తున్నారా?

ట్రయల్స్ మధ్య అవసరమైన వాష్ అవుట్ పీరియడ్‌లకు మీరు కట్టుబడి ఉన్నారా? ఉండకపోతే, మీ నిర్ణయాన్ని ప్రభావితం చేసింది ఏమిటి? (పరిశీలన: ఆర్థిక కారణాలు, ఏజెంట్ ప్రభావం, తోటివారి ఒత్తిడి?)

మీరు ట్రయల్స్ లో పాల్గొన్నప్పుడు లేదా తరువాత ఏదైనా స్వల్పకాలిక లేదా దీర్ఘకాలిక ఆరోగ్య సమస్యలను ఎదుర్కొన్నారా? (పరిశీలన: దుష్ప్రభావాలు) ఈ ఆరోగ్య సమస్యలను మీరు ఎలా ఎదుర్కొన్నారు?

మీరు ట్రయల్స్‌లో పాల్గొన్నప్పుడు లేదా తరువాత ఎప్పుడైనా మానసికంగా లేదా భావోద్వేగానికి గురయ్యారా? ఈ భావాలను మీరు ఎలా ఎదుర్కొన్నారు?

మీరు చికిత్స పొందగలిగారా?

మీకు ఏదైనా పరిహారం అందుకున్నారా? క్లినికల్ ట్రయల్స్ నుండి పొందిన చెల్లింపులు మీ రోజువారీ జీవితాన్ని ఎలా ప్రభావితం చేశాయి? (పరిశీలన: ఇది సరిపోతుందా? ప్రాథమిక మనుగడ అవసరాలను తీర్చిందా?)

మీకు ఉన్న పూర్తి అనుభవం ద్వారా, అటువంటి క్లినికల్ ట్రయల్స్ కోసం వాలంటీర్ గా ఉండటం ఎలా అనిపించింది?

మీరు పాల్గొనడం వల్ల మీకు ఏవైనా మంచి విషయాలు జరిగాయని మీరు అనుకుంటున్నారా? (పరిశీలన: అవి ఏమిటి? మీకు ఎలా అనిపించింది? అది మీ జీవితాన్ని ఏ విధంగానైనా మెరుగుపరిచిందా?)

మీరు పాల్గొనడం వల్ల మీకు ఏదైనా చెడు విషయాలు జరిగాయి అని మీరు అనుకుంటున్నారా? (పరిశీలన: అది ఎందుకు చెడ్డది? మీరు దానిని ఎలా ఎదుర్కొన్నారు?)

ప్రమాదాలు ఉన్నప్పటికీ మీ కమ్యూనిటీకి చెందిన వ్యక్తులు ట్రయల్స్‌లో పాల్గొనడం కొనసాగిస్తారని మీరు భావిస్తున్నారా? ఎందుకు లేదా ఎందుకు కాదు?

మీ అనుభవం గురించి మీరు ఇంకా ఏవైనా వివరాలు పంచుకోవాలనుకుంటున్నారా?

ఈ ఇంటర్వ్యూను మీరు ఎలా కనుగొన్నారు?

మీ అనుభవాలను నాతో పంచుకున్నందుకు ధన్యవాదాలు. మీ అభిప్రాయాలు చాలా విలువైనవి మరియు సీరియల్ క్లినికల్ ట్రయల్‌లో పాల్గొనడం యొక్క నిజానిజాలు అర్థం చేసుకోవడంలో మాకు సహాయపడతాయి.

# **Supplementary Material S2**

**In-Depth Interview Guide for key informants of persons who have taken part in multiple clinical trials (in English)**

Thank you for agreeing to participate. I want to assure you that I am not a doctor and have no connection with pharmaceutical companies or health authorities. This study aims to understand the experiences of individuals who have participated in multiple clinical trials. I am interested in finding out from you what your good and bad experiences have been living with a person who had taken part in multiple clinical trials, and there are no right or wrong answers.

Your responses will remain strictly confidential, and your name will never be revealed. You may stop the interview at any time.

Can you tell me a bit about yourself? (Age, education, occupation, family background)

How long you have been living in this village? What does this community mean to you?

When was the first time you found out that XXX was taking part in clinical trials?

For how long has XXX been participating in clinical trials?

How many times has XXX taken part in the such clinical trials?

What were the reasons for XXX to participate in subsequent clinical trials?

Did XXX discuss about his decision to take part in clinical trials with anyone in the family or others? (Probe: Spouse, parent, sibling, children, friend, doctor)

How did XXX first hear about clinical trials? (Probe: Friend, WhatsApp group, mobile text messages, agent, pharma company advert, doctor?)

How did his participation affect relationships with family and community members?

How did your family react when they found out about XXX’s participation in trials?

Did you notice any changes in XXX’s health, mood, or behaviour after participating in trials?

Did XXX seek medical help? (Probe: From where?)

What were the biggest challenges your family faced because of XXX’s participation in trials?

Looking back, do you wish you had done something differently regarding XXX’s participation in clinical trials?

Do you think people from your community will continue participating in trials despite the risks? Why or why not?

Is there anything else you would like to tell me?

Thank you for sharing your experiences with me. Your insights are valuable and will help in understanding the realities of serial clinical trial participation.

**In-Depth Interview Guide for key informants of persons who have taken part in multiple clinical trials (in Telugu)**

**బహుళ క్లినికల్ ట్రయల్స్‌లో పాల్గొన్న వ్యక్తుల యొక్క కీలక సమాచారం అందించే వ్యక్తుల కొరకు సవివరమైన ఇంటర్వ్యూ గైడ్ (తెలుగులో)**

ఈ కార్యక్రమంలో పాల్గొనడానికి అంగీకరించినందుకు ధన్యవాదాలు. నేను వైద్యుడిని కాదని, నాకు ఫార్మాస్యూటికల్ కంపెనీలు లేదా ఆరోగ్య అధికారులతో ఎటువంటి సంబంధం లేదని మీకు భరోసా ఇవ్వాలనుకుంటున్నాను. బహుళ క్లినికల్ ట్రయల్స్‌ లో పాల్గొన్న వ్యక్తుల అనుభవాలను అర్థం చేసుకోవడమే ఈ అధ్యయనం ముఖ్య లక్ష్యం. బహుళ క్లినికల్ ట్రయల్స్‌లో పాల్గొన్న వ్యక్తితో మీకు గల మంచి మరియు చెడు అనుభవాలు తెలుసుకోవడానికి నేను ఆసక్తిగా ఉన్నాను మరియు ఇందులో సరైన లేదా తప్పు సమాధానాలు లేవు.

మీ ప్రతిస్పందనలు ఖచ్చితంగా గోప్యంగా ఉంటాయి మరియు మీ పేరు ఎప్పటికీ వెల్లడించబడదు. మీరు ఏ సమయంలోనైనా ఇంటర్వ్యూను ఆపివేయవచ్చు.

మీ గురించి కొన్ని వివరాలు చెప్పగలరా? (వయస్సు, చదువు, వృత్తి, కుటుంబ నేపథ్యం)

మీరు ఈ గ్రామంలో ఎంతకాలంగా నివసిస్తున్నారు? ఈ కమ్యూనిటీ మీకు ఎంత ముఖ్యం?

XXX క్లినికల్ ట్రయల్స్‌లో పాల్గొంటున్నట్లు మీరు మొదటిసారిగా ఎప్పుడు కనుగొన్నారు?

XXX ఎంతకాలంగా క్లినికల్ ట్రయల్స్‌లో పాల్గొంటున్నారు?

XXX అటువంటి క్లినికల్ ట్రయల్స్‌లో ఎన్నిసార్లు పాల్గొన్నారు?

XXX తదుపరి క్లినికల్ ట్రయల్స్‌లో పాల్గొనడానికి కారణాలు ఏమిటి?

క్లినికల్ ట్రయల్స్‌లో పాల్గొనాలన్న తన నిర్ణయం గురించి XXX కుటుంబంలో ఎవరితోనైనా లేదా ఇతరులతోనైనా చర్చించారా? (పరిశీలన: జీవిత భాగస్వామి, తల్లిదండ్రులు, తోబుట్టువులు, పిల్లలు, స్నేహితుడు, వైద్యుడు)

క్లినికల్ ట్రయల్స్ గురించి XXX మొదట ఎలా కనుగొన్నారు? (పరిశీలన: స్నేహితుడు, వాట్సప్ గ్రూప్, మొబైల్ టెక్స్ట్ మెసేజ్‌లు, ఏజెంట్, ఫార్మా కంపెనీ ప్రకటన, డాక్టర్?)

అతని భాగస్వామ్యం కుటుంబం మరియు కమ్యూనిటీ సభ్యులతో సంబంధాలను ఎలా ప్రభావితం చేసింది?

XXX ట్రయల్స్‌లో పాల్గొనడం గురించి తెలుసుకున్నప్పుడు మీ కుటుంబం ఏ విధంగా స్పందించింది?

XXX ట్రయల్స్ లో పాల్గొన్న తరువాత వారి ఆరోగ్యం, మానసిక స్థితి లేదా ప్రవర్తనలో ఏవైనా మార్పులను మీరు గమనించారా?

XXX వైద్య సహాయం కోరారా? (పరిశీలన: ఎక్కడి నుంచి?)

XXXట్రయల్స్‌లో పాల్గొనడం వల్ల మీ కుటుంబం ఎదుర్కొన్న అతిపెద్ద సవాళ్లు ఏమిటి?

గతంలోకి చూస్తే, క్లినికల్ ట్రయల్స్ లో XXX యొక్క భాగస్వామ్యానికి సంబంధించి మీరు భిన్నంగా ఏదైనా చేసి ఉంటే బాగుండేదని మీరు అనుకుంటున్నారా?

ప్రమాదాలు ఉన్నప్పటికీ మీ కమ్యూనిటీకి చెందిన వ్యక్తులు ట్రయల్స్‌లో పాల్గొనడం కొనసాగిస్తారని మీరు భావిస్తున్నారా? ఎందుకు లేదా ఎందుకు కాదు?

మీరు నాకు ఇంకేమైనా వివరాలు చెప్పాలనుకుంటున్నారా?

మీ అనుభవాలను నాతో పంచుకున్నందుకు ధన్యవాదాలు. మీ అభిప్రాయాలు చాలా విలువైనవి మరియు సీరియల్ క్లినికల్ ట్రయల్‌లో పాల్గొనడం యొక్క నిజానిజాలను అర్థం చేసుకోవడంలో సహాయపడతాయి.

# **Supplementary Table S3**

COREQ (COnsolidated criteria for REporting Qualitative studies): 32-item checklist

| **Topic** | **Item**  **No.** | **Description** | **Reported on Page No.** | **Section** |
| --- | --- | --- | --- | --- |
| **Domain 1: Research team and reflexivity** | | | | |
| **Personal characteristics** | | | | |
| Interviewer/ facilitator | 1 | Which author/s conducted the interview or focus group? | 4 | Ethical considerations and Data collection |
| Credentials | 2 | What were the researcher's credentials? *E.g. PhD, MD* | 5 | Researchers’ characteristics, positionality, and reflexivity |
| Occupation | 3 | What was their occupation at the time of the study? | 5 | Researchers’ characteristics, positionality, and reflexivity |
| Gender | 4 | Was the researcher male or female? | 5 | Researchers’ characteristics, positionality, and reflexivity |
| Experience and  training | 5 | What experience or training did the researcher have? | 5 | Researchers’ characteristics, positionality, and reflexivity |
| **Relationship with participants** | | | | |
| Relationship  established | 6 | Was a relationship established prior to study commencement? | 3 | Study setting and Participant approach |
| Participant knowledge of the interviewer | 7 | What did the participants know about the researcher? *E.g. Personal goals, reasons for doing the research* | 3 | Study setting and Participant approach |
| Interviewer characteristics | 8 | What characteristics were reported about the interviewer/facilitator? *E.g. Bias, assumptions, reasons and interests in the research topic* | 5 | Researchers’ characteristics, positionality, and reflexivity |
| **Domain 2: Study design** | | | | |
| **Theoretical framework** | | | | |
| Methodological orientation and theory | 9 | What methodological orientation was stated to underpin the study? *E.g. grounded theory, discourse analysis, ethnography, phenomenology, content analysis* | 3 | Qualitative approach |
| **Participant selection** | | | | |
| Sampling | 10 | How were participants selected? *E.g. purposive, convenience, consecutive, snowball* | 4 | Sampling and Participant recruitment |
| Method of approach | 11 | How were participants approached? *E.g. face-to-face, telephone, mail, email* | 3 | Study setting and Participant approach |
| Sample size | 12 | How many participants were in the study? | 4 | Sampling and Participant recruitment |
| Non-participation | 13 | How many people refused to participate or dropped out? What were the reasons for this? | 4 | Sampling and Participant recruitment |
| **Setting** | | | | |
| Setting of data  collection | 14 | Where was the data collected? *E.g. home, clinic, workplace* | 3 | Study setting and Participant approach |
| Presence of non-  participants | 15 | Was anyone else present besides the participants and researchers? | 4 | Ethical considerations and Data collection |
| Description of sample | 16 | What are the important characteristics of the sample? *E.g. demographic data, date* | 7 | Participant characteristics |
| **Data collection** | | | | |
| Interview guide | 17 | Were questions, prompts, guides provided by the authors? Was it pilot tested? | S1, S2 | Supplement |
| Repeat interviews | 18 | Were repeat interviews carried out? If yes, how many? | NA | Not applicable |
| Audio/visual recording | 19 | Did the research use audio or visual recording to collect the data? | NA | Not applicable |
| Field notes | 20 | Were field notes made during and/or after the interview or focus group? | 4 | Ethical considerations and Data collection |
| Duration | 21 | What was the duration of the interviews or focus group? | 4 | Ethical considerations and Data collection |
| Data saturation | 22 | Was data saturation discussed? | 4 | Sampling and Participant recruitment |
| Transcripts returned | 23 | Were transcripts returned to participants for comment and/or correction? | NA | Not applicable |
| **Domain 3: Analysis and findings** | | | | |
| **Data analysis** | | | | |
| Number of data  coders | 24 | How many data coders coded the data? | 5 | Data analysis |
| Description of the  coding tree | 25 | Did authors provide a description of the coding tree? | 6 | Results |
| Derivation of themes | 26 | Were themes identified in advance or derived from the data? | 6 | Results |
| Software | 27 | What software, if applicable, was used to manage the data? | NA | Not applicable |
| Participant checking | 28 | Did participants provide feedback on the findings? | NA | Not applicable |
| **Reporting** | | | | |
| Quotations presented | 29 | Were participant quotations presented to illustrate the themes / findings? Was each quotation identified? *E.g. Participant number* | 6 | Results |
| Data and findings  consistent | 30 | Was there consistency between the data presented and the findings? | 6 | Results |
| Clarity of major themes | 31 | Were major themes clearly presented in the findings? | 6 | Results |
| Clarity of minor  themes | 32 | Is there a description of diverse cases or discussion of minor themes? | 6 | Results |

COREQ Checklist developed from Tong, A., Sainsbury, P., & Craig, J. (2007). Consolidated criteria for reporting qualitative research (COREQ): a 32-item checklist for interviews and focus groups. International Journal for Quality in Health Care, 19(6), 349–357. <https://doi.org/10.1093/intqhc/mzm042> [17]
